# An exploratory integrative analysis of plasma transcriptomic and proteomic predictors of response to total neoadjuvant therapy in locally advanced rectal cancer

**DOI:** 10.3389/fonc.2026.1799004

**Published:** 2026-06-08

**Authors:** Zonglin Zhu, Kaizhen Wen, Baoyi Zou, Yanhua Kang, Jingqing Chen, Bin Zhu, Liping Fan, Haobo Huang

**Affiliations:** 1 Medical Laboratory Center, Jinjiang Municipal Hospital (Shanghai Sixth People’s Hospital Fujian), Quanzhou, Fujian, China; 2 Department of Blood Transfusion, Fujian Medical University Union Hospital, Fuzhou, Fujian, China; 3 Department of Laboratory Medicine, Fujian Medical University Union Hospital, Fuzhou Fujian, China

**Keywords:** locally advanced rectal cancer, pathologic complete response, plasma, predictor, proteomics, total neoadjuvant therapy, transcriptomics

## Abstract

**Background:**

LARC patients exhibit heterogeneity in their response to total neoadjuvant therapy (TNT). This study aims to screen and identify plasma biomarkers associated with treatment response to TNT in patients with locally advanced rectal cancer (LARC) to predict pathological complete response (pCR).

**Methods:**

Pre-treatment plasma RNA sequencing and functional annotation were performed in six patients with LARC (three pCR and three non-pCR) to identify differentially expressed genes (DEGs) associated with TNT response. The Olink^®^ Target 96 Oncology II panel was employed to validate a subset of DEGs at the protein level in 61 patients with LARC (20 pCR vs 41 non-pCR), 6 patients with rectal adenoma (RA), and 6 healthy participants. Correlations between pretreatment plasma differentially expressed proteins (DEPs), clinicopathological characteristics, and TNT-response in LARC patients were analyzed.

**Results:**

Compared to the non-pCR group, 365 DEGs were upregulated, and 198 were downregulated in the pCR group. These genes are involved in pathways related to cell growth and death, signal transduction, metabolism, the immune system, insulin secretion, mitophagy, and neutrophil extracellular trap formation. The transcriptomic analysis was conducted in a limited discovery cohort, and these DEGs identified should be interpreted with extreme caution due to the high risk of false discovery. Plasma levels of Fas ligand (FASLG), CD160, andLY6/PLAUR domain containing 3 (LYPD3) in the pCR group were higher than those in the non-pCR group. Receiver operating characteristic (ROC) curve analysis showed that a multi-marker panel consisting of FASLG, CD160, and LYPD3 was superior to three individual indicators in predicting pCR, with an area under the curve (AUC) of 0.791 (95% CI, 0.668-0.885). According to the optimal cutoff value of the multi-marker panel, all LARC patients were stratified into high (n=34, 55.7%) and low (n=27, 44.3%) expression groups. Statistical analysis indicated that high expression group was associated with a higher proportion of EMVI-negative status and a higher pCR rate. High expression of multi-marker was associated with pCR in multivariate analysis.

**Conclusions:**

The multi-marker panel consisting of FASLG, CD160, and LYPD3 may serve as a candidate predictor of treatment response in LARC patients undergoing TNT, warranting further validation in larger, multicenter cohorts.

## 1 Introduction

Colorectal cancer (CRC) remains one of the most common cancers worldwide. Approximately 40% of the patients have rectal cancer (RC), of which locally advanced rectal cancer (LARC) accounts for over half of all reported cases at initial diagnosis. The current treatment for LARC is neoadjuvant chemoradiotherapy (nCRT) followed by total mesorectal excision (TME). However, several studies have revealed that total neoadjuvant therapy (TNT) can improve disease-free survival (DFS) and overall survival (OS) benefits for patients with LARC, as well as increase organ preservation rates (1–4). Among these patients, some achieve clinical complete response (cCR) following TNT and are recommended to receive the “Watch and Wait” (W&W) strategy (5–7). Nevertheless, a few patients were subsequently confirmed to have not achieved pathological complete response (pCR) after TME (8). Therefore, achieving a more accurate preoperative prediction of pCR after TNT has significant clinical implications for patients with LARC, enabling organ preservation, reducing surgical complications, and enhancing quality of life. However, no precise biomarkers currently enable the reliable preoperative prediction of pCR.

Plasma transcriptomics, either independently or in combination with proteomic analyses, has demonstrated significant efficacy in biomarker screening for early cancer detection or treatment response evaluation (9–15). However, few studies have reported its application in predicting treatment response to TNT in LARC.

Therefore, in this study, we integrated plasma transcriptomic and proteomic profiles from patients with LARC prior to TNT, with pathological outcomes following TME to identify circulating biomarkers associated with the TNT response. This comprehensive biomarker analysis provides a valuable supplementary approach for predicting pCR prior to TME.

## 2 Materials and methods

### 2.1 Patients

From January 2022 to December 2024, 73 blood samples were collected from 61 patients with LARC prior to TNT, 6 patients with rectal adenoma (RA), and 6 healthy participants who underwent routine medical examination. Pathological diagnoses of all patients with LARC and RA were confirmed by two pathologists. Magnetic resonance imaging (MRI) was used for abdominal evaluation, and computed tomography (CT) was used for screening for systemic metastases. Diagnostic criteria were based on the American Joint Committee on Cancer (AJCC) 8th Edition guidelines, defining LARC as adenocarcinoma with the following conditions confirmed by MRI either:(1) clinical T3/T4 N0 lesions located within 15 cm of the anal verge as confirmed by endoscopic evaluation, or (2) any T-stage with nodal involvement (N1/N2). Eligibility criteria comprised patients aged 18 years or older with confirmed stage II–III rectal cancer who completed TNT involving radiotherapy combined with four cycles of CAPOX regimen chemotherapy, prior to curative resection. Eligible participants needed to have an ECOG performance status of 0–1 and demonstrate sufficient hematological, hepatic, and renal function. Exclusion criteria comprised participants presenting with recurrent or metastatic malignancies or those with previous exposure to antitumor therapies. Consolidation chemotherapy was administered in 21-day cycles. Each 21-day cycle included intravenous oxaliplatin (130 mg/m² over 2 h on day 1) and oral capecitabine (1000 mg/m² twice daily from days 1 to 14). All patients with LARC received either long-course radiotherapy (total dose of 50.4 Gy, delivered in 28 fractions) or short-course radiotherapy (total dose of 25 Gy, delivered in 5 daily fractions of 5 Gy). All patients with LARC underwent TME within 28 days following completion of TNT at Fujian Medical University Union Hospital. Pathological assessment was conducted by experienced pathologists. pCR was defined as the complete absence of tumor cells in both the primary site and lymph nodes (ypT0N0) following curative resection.

### 2.2 Blood sample collection and processing

Blood samples were collected from patients with LARC before TNT. Blood samples from patients with RA and healthy participants were collected concurrently during a medical examination in an EDTA-containing sterile vacutainer tube and centrifuged at 4°C and 3, 000 rpm for 10 min. Plasma of each blood sample was stored at −80°C.

### 2.3 Plasma RNA sequencing and data processing

Total RNA was extracted from 1 mL of plasma using a plasma/serum miRNA ex-traction kit (Bio You). The concentration of the extracted total RNA was assessed using a Qubit RNA assay (Life Technologies). A total RNA library (>50 nt) was prepared using a SMARTer Stranded Total RNA-Seq Kit v2.

Following the kit protocol, reverse transcription, addition of sequencing adapters and barcodes, construction and purification of cDNA libraries, depletion of ribosomal RNA (rRNA), PCR amplification of cDNA libraries, and additional purification steps were performed. Purified cDNA libraries were sequenced using an Illumina Nova-Seq6000 platform.

Raw sequencing data were quality-trimmed to remove adapter contamination and low-quality ends using Seqtktrimfq v1.0, thereby generating clean reads for subsequent analysis. Quality control of the clean reads was performed using fastQC (FastQC v0.10.1) The reads were aligned to the reference genome (GRCh38.p14) using Hisat2 (v2.0.4) with default parameters to generate BAM files for downstream analysis. The number of fragments mapped to each gene after alignment by Hisat2 was counted by Stringtie (version: 1.3.0). Then normalization was performed with the TMM (trimmed mean of M values) method. To quantify gene expression, FPKM (Fragments Per Kilobase of the exon model per million mapped fragments) values for each gene were calculated using a Perl script.

### 2.4 Differential analysis and functional enrichment analysis of plasma RNA sequencing data

The edge R package (v3.2.0) was used to identify differentially expressed genes (DEGs) (|log2[fold-change]|> 1 and P value <0.05). Gene Ontology (GO), Kyoto Encyclopedia of Genes and Genomes (KEGG) functional enrichment, and gene set enrichment analyses (GSEA) were performed to determine the functional categories of the DEGs. The transcriptomic discovery was performed in only 6 patients (3 vs. 3) and that the number of DEGs should be interpreted with extreme caution due to the high risk of false discovery.

### 2.5 Plasma proteins detection by Olink assays and differential analysis

Plasma samples from LARC patients were thawed and analyzed using the Olink^®^ Target 96 Oncology II panel (Olink Proteomics AB, Uppsala, Sweden), performed by the Shanghai Biotechnology Corporation according to the manufacturer’s instructions.

Briefly, target proteins in the plasma are recognized by pairs of antibodies conjugated with unique DNA oligonucleotide labels. If pairs of antibodies are brought into proximity, the oligonucleotides hybridize, are recognized, and extended by DNA poly-merase. The oligonucleotides were pre-amplified by PCR, followed by real-time PCR quantification, thereby enabling precise quantification of target proteins. Real-time PCR data were preprocessed using the Olink NPX Signature software to generate NPX files.

Differentially expressed proteins (DEPs) were obtained using Student’s t-test, with a P-value cutoff of 0.05(P<0.05).

### 2.6 Statistical analysis

The chi-squared test or Fisher’s exact test was used to compare categorical variables. Unpaired t-tests or Mann-Whitney U tests were used to compare continuous variables. The binary logistic regression was used to construct a multi-marker prediction model. The regression parameters were fitted via the maximum likelihood estimation method. The Wald chi-square test was performed to test the statistical significance of the regression coefficient of each independent variable. The area under the curve (AUC) of the ROC curve was used to evaluate the predictive performance of the predictors. Statistical significance was defined as a two-sided P-value of < 0.05. A logistic regression model was used for the univariate analysis of potential predictors of response to TNT. The logistic regression model was used for multivariate analysis of predictors with P-values<0.05 in the univariate analysis. All statistical analyses were performed using the SPSS software (version 19.0; IBM Corp., Armonk, NY, USA) and GraphPad Prism (version 8.0; GraphPad Corp., Boston, USA).

## 3 Results

### 3.1 DEGs of plasma RNA between pCR and non-pCR groups

**Figure 1:**
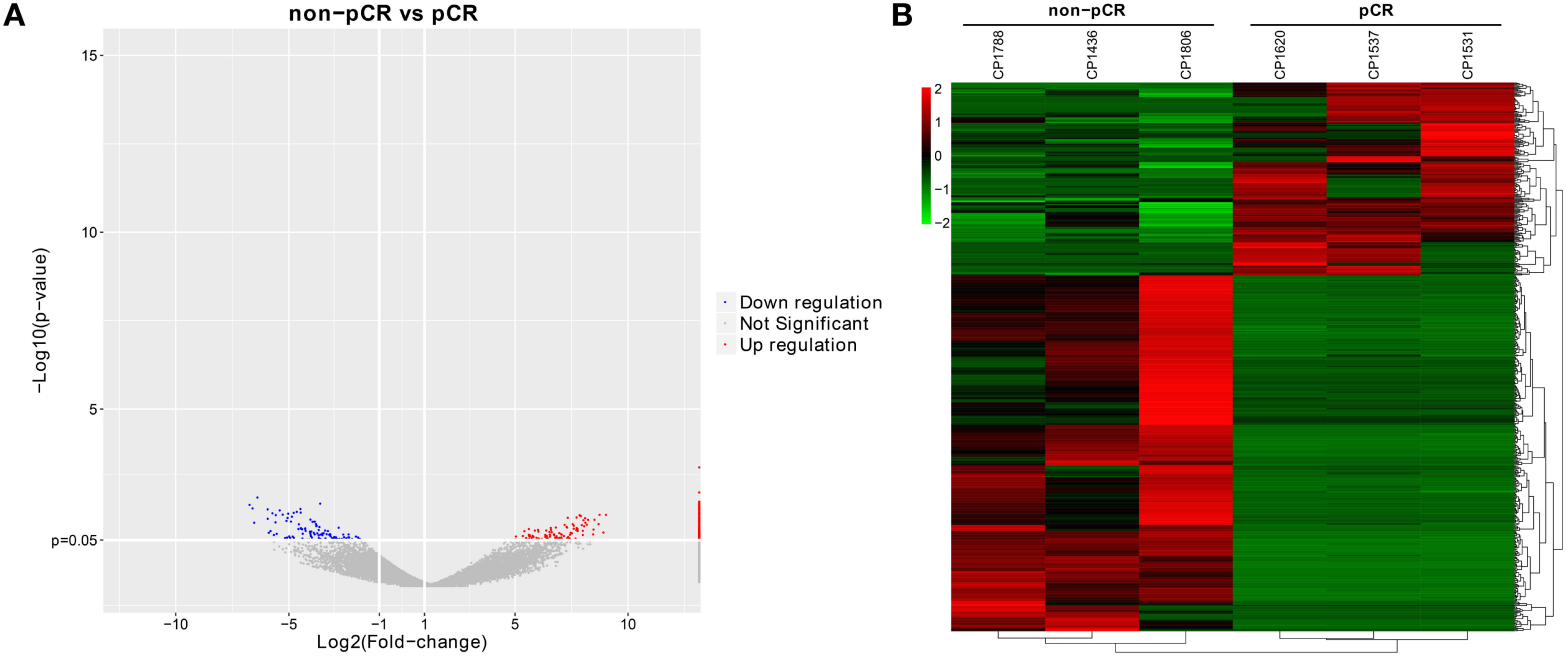
DEGs of plasma RNA between pCR and non-pCR groups. **(A)** Volcano plot; **(B)** heatmap.

We used plasma RNA sequencing to identify DEGs in LARC patients associated with TNT response. A total of 563 DEGs were associated with the TNT response. Compared to the non-pCR group, 365 DEGs were upregulated, and 198 were downregulated in the pCR group (**Figure 1**).

### 3.2 Functional enrichment analysis of DEGs in plasma

**Figure 2:**
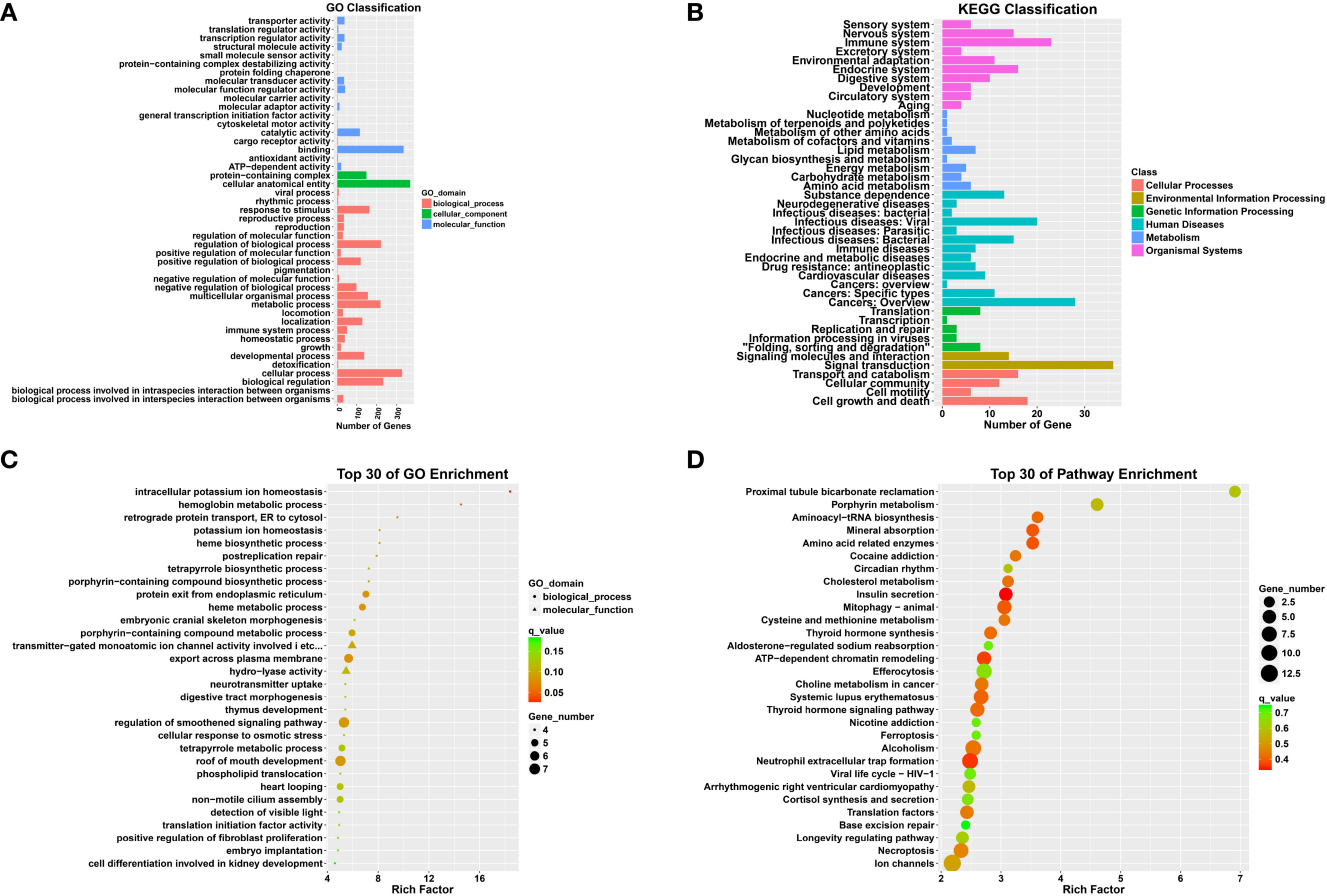
Functional annotation of DEGs. **(A)** GO (including BP, CC, and MF), **(B)** KEGG, **(C)** Top 30 of GO Enrichment, and **(D)** Top 30 of Pathway Enrichment.

GO classification showed that DEGs were involved in cellular processes, metabolic processes, and biological regulation, and exhibit many functions, including binding, catalytic activity, molecular function regulator activity, molecular transducer activity, and transcriptional regulator activity (**Figure 2A**). KEGG classification demonstrated that DEGs were involved in multiple pathways related to cancers, signal transduction, lipid metabolism, energy metabolism, amino acid metabolism, translation, transcription, DNA replication and repair, cell growth and death, and pathways related to the immune system (**Figure 2B**).

GO enrichment analysis indicated significant enrichments in pathways related to intracellular potassium ion homeostasis, hemoglobin metabolism, and retrograde protein transport from the ER to the cytosol. Pathways related to transmitter-gated monoatomic ion channels and hydrolase activity were significantly enriched (**Figure 2C**). KEGG enrichment analysis indicated that the pathways related to insulin secretion, mitophagy, and neutrophil extracellular trap formation were significantly enriched (**Figure 2D**).

### 3.3 DEPs of plasma protein and characteristics between pCR and non-pCR groups

**Figure 3:**
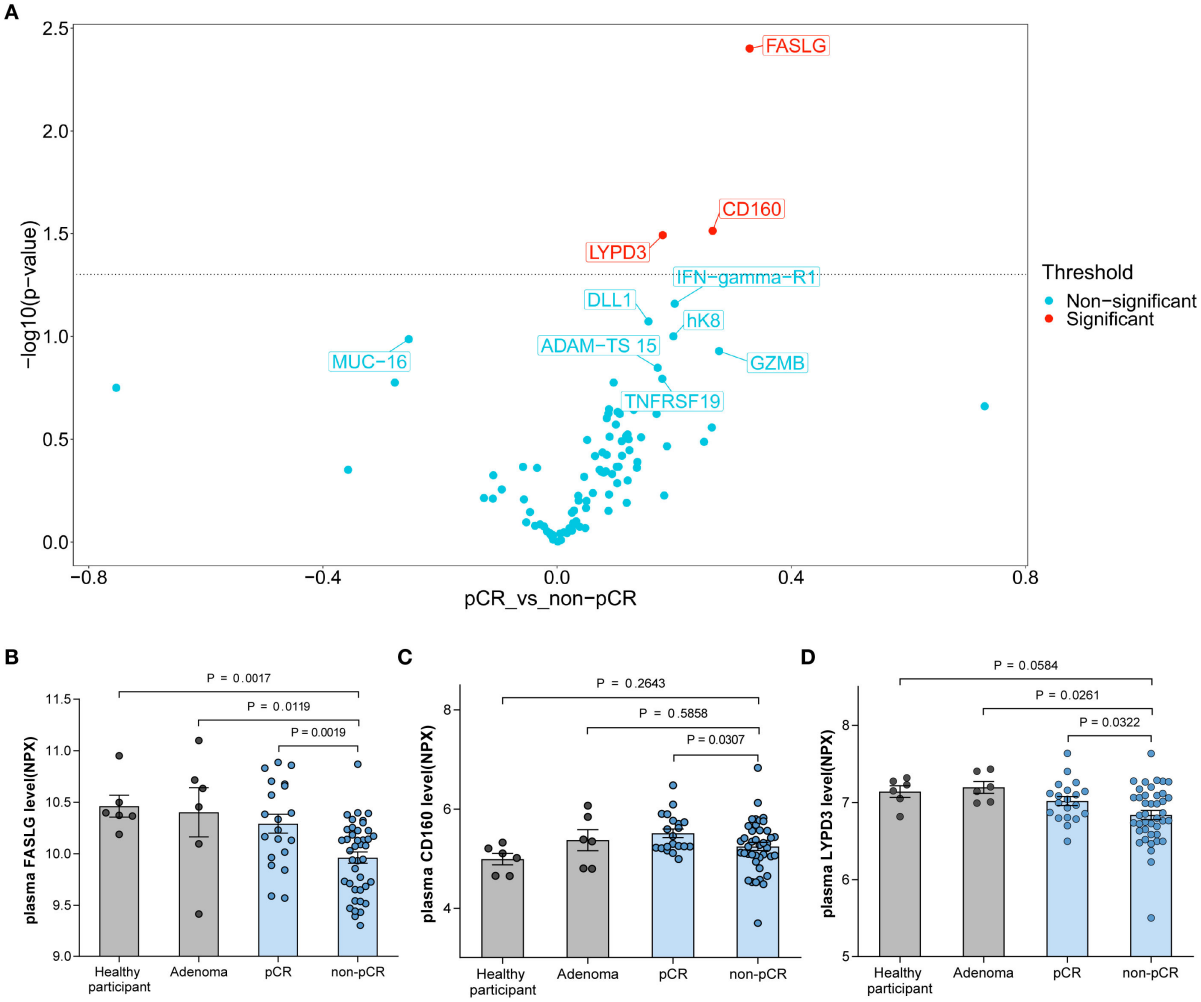
DEPs of plasma proteins between pCR and non-pCR groups. **(A)** Volcano plot;**(B–D)** Box plot with scatter points of FASLG, CD160, and LYPD3 in different groups.

Based on the classification and pathway enrichment analysis of DEGs of plasma RNA, we utilized the Olink^®^ Target 96 Oncology II panel to explore the plasma protein level of a subset of these DEGs in 61 LARC patients. The volcano plot demonstrates DEPs in the plasma between these two groups. Plasma levels of Fas ligand (FASLG), CD160, and LY6/PLAUR domain containing 3 (LYPD3) in the non-pCR group were lower than those in the pCR group (**Figure 3A**). Plasma FASLG levels in patients without pCR were also significantly lower than those in patients with RA and healthy participants. However, there was no statistically significant difference in plasma FASLG levels among pCR patients, RA patients, and healthy participants (**Figure 3B**) or in plasma CD160 levels among non-pCR patients, RA patients, and healthy participants. Plasma LYPD3 levels in non-pCR patients were lower than those in RA patients, but were not significantly different from those in healthy participants (**Figure 3D**).

Analysis of the differences in clinicopathological characteristics between the pCR and non-pCR groups showed that clinical T stage and EMVI status were significantly associated with pCR in patients with LARC (P<0.05). Treatment response was not significantly associated with sex, age, clinical N stage, MRF status, plasma CEA level, tumor location, or tumor size (**Table 1**).

**Table 1:** Comparison of clinicopathological characteristics and plasma biomarkers between the pCR and non-pCR groups.

Characteristic	pCR group (n=20)	Non-pCR group (n=41)	P value
Sex			0.953
Male	14 (70.0%)	29 (70.7%)	
Female	6 (30.0%)	12 (29.3%)	
Age (years)	58 (46-80)	64 (34-82)	0.958
Clinical T stage			0.012
T3	12 (60.0%)	11 (26.8%)	
T4	8 (40.0%)	30 (73.2%)	
Clinical N stage			0.623
N1	5 (25.0%)	8 (19.5%)	
N2	15 (75.0%)	33 (80.5%)	
EMVI status			0.035
Positive	8 (40.0%)	28 (68.3%)	
Negative	12 (60.0%)	13 (31.7%)	
MRF status			0.123
Positive	8 (40.0%)	25 (61.0%)	
Negative	12 (60.0%)	16 (39.0%)	
CEA (pg/ml)			0.781
High	9 (45.0%)	20 (48.8%)	
Normal	11 (55.0%)	21 (51.2%)	
Tumor location			0.061
low	13 (65.0%)	16 (39.0%)	
middle	7 (35.0%)	18 (43.9%)	
high	0 (0.0%)	7 (17.1%)	
Tumor size			0.279
≥5 cm	5 (25.0%)	16 (39.0%)	
<5 cm	15 (75.0%)	25 (61.0%)	
Radiotherapy			0.598
LCRT	19 (95.0%)	40 (97.6%) 6%)	
SCRT	1 (5.0%)	1 (2.4%)	
Plasma FASLG (NPX)	10.29 ± 0.4070	9.962 ± 0.3513	0.002
Plasma CD160 (NPX)	5.514 ± 0.3827	5.248 ± 0.5341	0.031
Plasma LYPD3 (NPX)	7.019 ± 0.2586	6.838 ± 0.3718	0.032

### 3.4 Association of plasma FASLG levels with clinicopathological characteristics and treatment response in LARC patients

**Figure 4:**
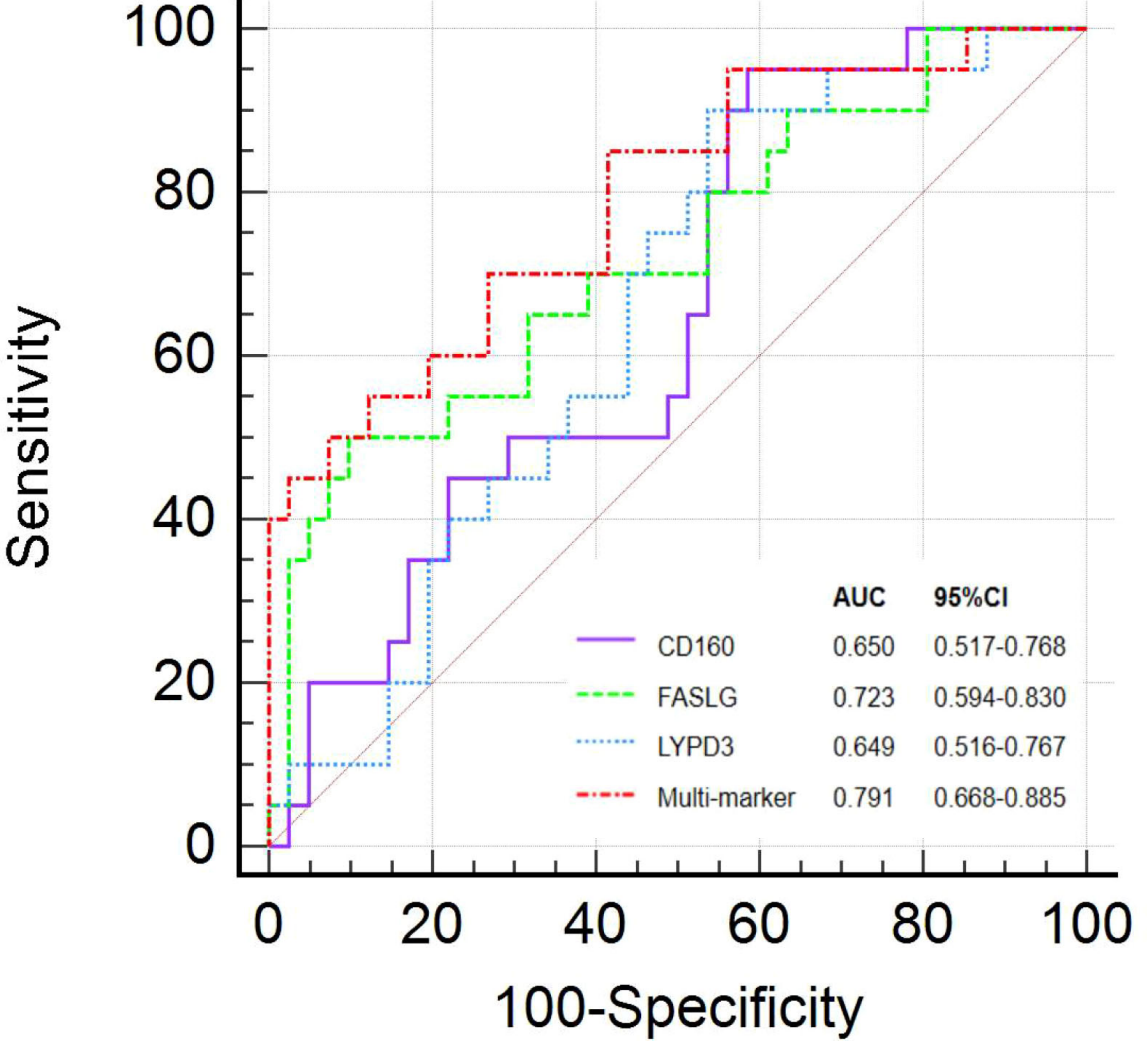
Receiver operating characteristic (ROC) curves of baseline plasma levels of FASLG, CD160, LYPD3 and a multi-marker panel for predicting TNT response.

Based on the treatment response information, ROC curve analysis was performed to evaluate the predictive value of the plasma levels of FASLG, CD160, LYPD3 and a multi-marker panel incorporating these three markers in distinguishing pCR patients from non-pCR patients. The final fitted logistic regression prediction equation of the multi-marker panel was: Y = −44.3830 + (2.3447 × LYPD3) + (2.2575 × FASLG) + (0.8419 × CD160). The areas under the curve for plasma levels of FASLG, CD160, LYPD3 and the multi-marker panel were 0.723 (95% confidence interval [CI]: 0.594–0.830, P = 0.005), 0.650(95% CI: 0.517–0.768, P = 0.059), 0.649(95% CI: 0.516–0.767, P = 0.061), and 0.791 (95% CI: 0.668–0.885, P < 0.001), respectively (**Figure 4**). Therefore, we employed the multi-marker panel for further analyses. According to the optimal cutoff value of the multi-marker panel (Y>0.2111), patients were stratified into high (n=34, 55.7%) and low (n=27, 44.3%) expression groups based on this panel. Further analysis indicated that high expression group was associated with a higher proportion of EMVI-negative status, and a higher pCR rate. There were no associations between this multi-marker panel and sex, age, clinical T stage, clinical N1 stage, MRF status, serum CEA levels, tumor location, or tumor size (**Table 2**).

**Table 2:** Association of the multi-marker panel with clinicopathological characteristics and treatment response in LARC patients.

Characteristic	High expression group (n=34)	Low expression group (n=27)	P value
Sex			0.397
Male	22(64.7%)	21(77.8%)	
Female	12(35.3%)	6(22.2%)	
Age(years)	63(34–82)	59(34–80)	0.991
Clinical T stage			0.115
T3	16(47.1%)	7(25.9%)	
T4	18(52.9%)	20(74.1%)	
Clinical N stage			0.534
N1	6(17.6%)	7(25.9%)	
N2	28(82.4%)	20(74.1%)	
EMVI status			0.010
Positive	15(44.1%)	21(77.8%)	
Negative	19(55.9%)	6(22.8%)	
MRF status			0.606
Positive	17(50.0%)	16(59.3%)	
Negative	17(50.0%)	11(40.7%)	
CEA (pg/ml)			0.800
High	15(44.1%)	13(48.1%)	
Normal	19(55.9%)	14(51.9%)	
Tumor location			0.277
low	18(52.9%)	11(40.7%)*	
middle	14(41.2%)	11(40.7%)*	
high	2(5.9%)	5(18.5%)*	
Tumor size			0.789
≥5cm	11(32.4%)	10(37.0%)	
<5cm	23(67.6%)	17(63.0%)	
Treatment response			0.002
pCR	17(50.0%)	3(11.1%)	
non-pCR	17(50.0%)	24(88.9%)	

*the mantissa of one digit is now the result. The mantissaof two digits adds up to 100%.

### 3.5 Univariate and multivariate analysis of predictors for pCR in LARC patients

In univariate analysis, clinical T3 stage, EMVI-negative status, and high expression of multi-marker were significantly associated with pCR, whereas sex, age ≥ 60 years, serum CEA level, clinical N stage, tumor location, MRF status, and tumor size were not significantly associated. The three statistically significant univariate factors (clinical T stage, EMVI status, and multi-marker) were incorporated into the multivariate analysis. Multivariate analysis suggested that clinical T3 stage and high expression of the multi-marker panel were independently associated with pCR in this cohort (**Table 3**).

**Table 3:** Univariate and multivariate analysis of predictors for pCR in LARC patients.

Variable	Univariate analysis HR (95% CI)	P value	Multivariate analysis HR (95% CI)	P value
Sex (Male vs. Female)	1.036(0.322–3.335)	0.953		
Age (≥60y vs. <60y)	2.118(0.713–6.293)	0.177		
CEA (high vs. low)	1.056(0.361–3.089)	0.921		
Clinical T stage (T3 vs. T4)	4.091(1.321–12.668)	0.015	3.589(1.023–12.591)	0.046
Clinical N stage (N1 vs. N2)	0.727(0.204–2.598)	0.624		
Tumor location (low vs. middle vs. high)		0.448		
Tumor size (≥5cm vs. <5cm)	1.920(0.584–6.317)	0.283		
MRF status (+ vs. –)	2.344(0.786–6.990)	0.127		
EMVI status (+ vs. –)	3.231(1.064–9.807)	0.038	2.239(0.620–8.084)	0.218
multi-marker (high vs. low)	0.125(0.032–0.495)	0.003	0.184(0.043–0.793)	0.023

## 4 Discussion

LARC is a common gastrointestinal tumor. Guidelines endorse nCRT followed by TME as a key therapeutic approach for LARC. Recent studies have shown that compared to nCRT, TNT can induce more sufficient tumor regression and improve the pCR rate (1–4). After TNT, some LARC patients who achieved cCR were recommended to the “W&W” strategy (5–7). Clinical studies have shown that lymph node metastasis may persist even in patients who achieve cCR, as LARC patients exhibit heterogeneity in their response to TNT. Therefore, the decision to use the “W&W” strategy for LARC patients who received TNT should be made with caution. Moreover, exploring potential predictive biomarkers of TNT response in patients with LARC is necessary, as it could minimize unnecessary surgery-related morbidity among those achieving pCR and prevent delayed surgery among those achieving cCR but not pCR.

Certain imaging parameters derived from positron emission tomography/computed tomography (PET/CT) or magnetic resonance imaging (MRI) can enhance the predictive proficiency for pCR in patients who received nCRT (16–19). However, these diagnostic modalities are subject to several limitations, including technical complexity and high cost. Therefore, it is critically important to identify readily available, reliable biological markers to accurately predict pCR.

In this study, we selected 61 consecutive LARC patients from our center during a defined timeframe as our research subjects. all of these patients met the inclusion criteria and did not meet the exclusion criteria. After receiving radiotherapy combined with four cycles of CAPOX regimen chemotherapy, 20 patients achieved pCR (20/61), with a pCR rate slightly higher than that reported in previous studies from our center (32.8% vs 28.3%) (20). We attributed this to the relatively small sample size and potential temporal bias in our study. The pCR rate in our study was also slightly higher than those reported in previous trials, such as PROSPECT (22%), RAPIDO (28%), PRODIGE23 (28%), and OPRA (25%) (1–4). As our study was a retrospective cohort study, patient selection bias, such as the exclusion of patients who could not tolerate 4 cycles of CAPOX due to treatment toxicity or who switched treatment regimens due to disease progression during therapy, also constituted an important factor contributing to this difference. Furthermore, this difference was likely attributable to several factors including the smaller sample size of our study compared to the aforementioned trials, the differential impact of LCRT and SCRT on pCR, as well as differences in chemotherapy regimens and the number of cycles administered on pCR.

Herein, we initially compared plasma DEGs between pCR and non-pCR LARC patients (3 pCR vs. 3 non-pCR) who received TNT and identified 365 upregulated and 198 downregulated DEGs in the pCR group. Then, we utilized the Olink^®^ Target 96 Oncology II panel to validate a subset of the aforementioned DEGs at the protein level in 61 LARC patients (20 pCR vs. 41 non-pCR).Ultimately, we confirmed that plasma levels of FASLG, CD160, and LYPD3 were significantly different between non-pCR and pCR patients with LARC. We also found that plasma FASLG can effectively predict non-pCR LARC patients in this cohort, as its levels were significantly lower than those in healthy participants, RA patients, and pCR LARC patients. However, plasma CD160 and LYPD3 did not demonstrate the same predictive ability. We suspected that the sample imbalance among healthy participants group, RA group, pCR group and non-pCR group may account for this phenomenon. Furthermore, comparative analysis of clinical characteristics indicated significant differences in clinical T stage and EMVI status between the pCR and non-pCR groups, consistent with previous findings.

Fas ligand (FASLG) is a member of the tumor necrosis factor superfamily and a transmembrane cytokine that is expressed in several cell types, including a subset of immune cells called mFASLG. The ectodomain of mFASLG can be cleaved by matrix metalloproteinase MMP3, MMP7, MMP9, disintegrin, and metalloproteinase (ADAM)-10 and released into the bloodstream as soluble FASLG (sFASLG). sFASLG has multiple biological functions and competitively antagonizes the death signals mediated by mFASLG, induces the synthesis of proinflammatory mediators, and triggers apoptotic signaling in tumor cells, thereby promoting phagocyte chemotaxis and enhancing their capacity to phagocytose and clear apoptotic cells. Furthermore, it can directly enhance the migratory ability of immune cells (21, 22). Plasma FASLG levels in LARC patients of pCR group were comparable to those in healthy participants, suggesting that the pro-apoptotic and anti-tumor immune functions mediated by FASLG remain relatively intact, and the immune microenvironment of these patients was not substantially suppressed. Furthermore, the lower plasma FASLG levels observed in LARC patients of non-pCR group indicated a potentially suppressed immune microenvironment, which may be linked to their treatment response. CD160 is a member of the immunoglobulin superfamily, primarily expressed on cytotoxic T lymphocytes, NK cells, and certain T-cell subsets. It is a multifunctional immunomodulatory molecule that can exert co-stimulatory or co-inhibitory functions (23, 24). However, the role and significance of soluble CD160 in plasma has not been reported. LYPD3 is a membrane-anchored protein that is highly expressed in various epithelial-derived tumor cells, such as lung cancer and colon cancer. It plays an important role in tumor invasion and metastasis by participating in cell adhesion and signal transduction, and is associated with poor prognosis in cancer patients (25–28). Similarly, the function and significance of soluble LYPD3 in plasma has not been reported. Our results at the protein level validated the findings of transcriptional analysis regarding the involvement of DEGs in cancer- and immune system-related pathways.

To explore the association between plasma levels of FASLG, CD160, and LYPD3 and both the clinical characteristics and treatment response in LARC patients, we performed ROC curve analysis to evaluate the predictive value of these individual markers and a multi-marker panel for pCR. The results indicated that the multi-marker panel had superior predictive value for pCR. We determined the optimal cut-off value of multi-marker panel using ROC curve analysis and divided the 61 patients who received TNT into high- and low-expression groups. Statistical analysis indicated that high expression group were significantly associated with a higher proportion of EMVI-negative status and a higher pCR rate. We also found that 88.9% (24/27) of LARC patients in low expression group did not achieve pCR. This observation raises the hypothesis that LARC patients with low expression of the multi-marker panel might represent a subgroup warranting investigation of alternative or intensified treatment strategies, though this requires prospective evaluation. Logistic regression analysis indicated that the multi-marker panel was independently associated with pCR. Moreover, clinical T stage was also independently associated with pCR, consistent with previous results (8, 29–31). Previous studies have shown that pretreatment plasma ctDNA, microRNA panel, and immune signatures can effectively predict treatment response to TNT in LARC patients (32–35). Our results indicated that the performance of the multi-marker in distinguishing treatment response (pCR/non-pCR) is similar to that of predictive models reported previously, with comparable AUC values. However, variations persisted in the sensitivity and specificity across these models, indicating that the pursuit of more efficient predictive indicators continues to be of value.

In our study, all patients with LARC received a neoadjuvant treatment regimen that combined CAPOX chemotherapy (oxaliplatin and capecitabine) and LCRT. Both oxaliplatin and radiotherapy can induce immunogenic cell death (ICD) in tumor cells, thereby activating the body’s immune system and mediating antitumor immune responses (36–40). Our study demonstrated at the transcriptional level that TNT treatment response in LARC patients is associated with immune system-related and cancer-related pathways. At the protein level, we further identified that TNT treatment response in LARC patients is also associated with FASLG- and CD160-related immune system pathways, as well as LYPD3-associated cancer pathways. These results indicate that for LARC patients, the function of immune system plays a role in their response to CAPOX plus LRCT treatment.

Although our study demonstrated that the multi-marker panel consists of FASLG, CD160 and LYPD3 was independently associated with pCR in patients with LARC who received TNT, several limitations remain. First, the small sample size in the discovery stage resulted in a high risk of false discoveries in DEG screening. Second, the incomplete validation of DEGs resulted in the failure to identify molecules with higher predictive performance. Third, in the validation stage, the small sample size of our cohort and the lack of external cohort validation have limited the generalizability and clinical application of our conclusions. Future studies should consider increasing the sample size in the discovery and validation stage, expanding the range of validation indicators, incorporating multiomics factors, and exploring additional biomarkers to enhance the predictive efficacy.

## 5 Conclusions

The multi-marker panel consisting of FASLG, CD160, and LYPD3 showed promise as a potential predictor of pCR in LARC patients undergoing TNT in this exploratory analysis. Furthermore, among the DEGs not validated at the protein level in this study, there may still exist molecules with promising predictive efficacy for pCR. External validation in larger, multicenter prospective cohorts is necessary before clinical application can be considered.

## Data Availability

The data presented in the study are deposited in the Gene Expression Omnibus (GEO) repository, accession number GSE333629.

## References

[1] Bahadoer RR Dijkstra EA van Etten B Marijnen CAM Putter H Kranenbarg EM . RAPIDO: a randomised, open-label, phase 3 trial. Lancet Oncol. (2021) 22:29–42. doi: 10.1016/s1470-2045(20)30555-6 33301740

[2] Basch E Dueck AC Mitchell SA Mamon H Weiser M Saltz L . The PROSPECT trial (Alliance N1048). J Clin Oncol Off J Am Soc Clin Oncol. (2023) 41:3724–34. doi: 10.1200/jco.23.00903 37270691 PMC10351948

[3] Conroy T Bosset JF Etienne PL Rio E Francois E Mesgouez-Nebout N . (UNICANCER-PRODIGE 23): a multicentre, randomised, open-label, phase 3 trial. Lancet Oncol. (2021) 22:702–15. doi: 10.1016/s1470-2045(21)00079-6 33862000

[4] Garcia-Aguilar J Patil S Gollub MJ Kim JK Yuval JB Thompson HM . OPRA: Organ preservation in patients with rectal adenocarcinoma treated with total neoadjuvant therapy. J Clin Oncol Off J Am Soc Clin Oncol. (2022) 40:2546–56. doi: 10.1200/jco.22.00032 35483010 PMC9362876

[5] Cerdan-Santacruz C Sao Juliao GP Vailati BB Corbi L Habr-Gama A Perez RO . Watch and wait approach for rectal cancer. J Clin Med. (2023) 12:2873. doi: 10.3390/jcm12082873 37109210 PMC10143332

[6] Huisman JF Schoenaker IJH Brohet RM Reerink O van der Sluis H Moll FCP . Avoiding unnecessary major rectal cancer surgery by implementing structural restaging and a watch-and-wait strategy after neoadjuvant radiochemotherapy. Ann Surg Oncol. (2021) 28:2811–8. doi: 10.1245/s10434-020-09192-0 33170456 PMC8043907

[7] Meng C Shu W Sun L Wu S Wei P Gao J . Rectal cancer approach strategies after neoadjuvant treatment - a systematic review and network meta-analysis. Int J Surg. (2025) 111:3078–92. doi: 10.1097/js9.0000000000002290 39945776 PMC12175800

[8] Zhong X Zeng G Zhang L You S Fu Y He W . Prediction of pathologic complete response to neoadjuvant chemoradiation in locally advanced rectal cancer. Front Oncol. (2024) 14:1361300. doi: 10.3389/fonc.2024.1361300 38529385 PMC10961458

[9] Kartsonaki C Pang Y Millwood I Yang L Guo Y Walters R . Circulating proteins and risk of pancreatic cancer: a case-subcohort study among Chinese adults. Int J Epidemiol. (2022) 51:817–29. doi: 10.1093/ije/dyab274 35064782 PMC9189974

[10] Liu J Wang Y Tian Z Lin Y Li H Zhu Z . Multicenter phase II trial of Camrelizumab combined with Apatinib and Eribulin in heavily pretreated patients with advanced triple-negative breast cancer. Nat Commun. (2022) 13:3011. doi: 10.1038/s41467-022-30569-0 35641481 PMC9156739

[11] Tang Z Gu Y Shi Z Min L Zhang Z Zhou P . Multiplex immune profiling reveals the role of serum immune proteomics in predicting response to preoperative chemotherapy of gastric cancer. Cell Rep Med. (2023) 4:100931. doi: 10.1016/j.xcrm.2023.100931 36724786 PMC9975277

[12] Xu F Xu H Wan Z Yang G Yang L Wu X . A linear discriminant analysis model based on the changes of 7 proteins in plasma predicts response to Anlotinib therapy in advanced non-small cell lung cancer patients. Front Oncol. (2021) 11:756902. doi: 10.3389/fonc.2021.756902 35070967 PMC8777128

[13] Yang X Suo C Zhang T Yin X Man J Yuan Z . Targeted proteomics-derived biomarker profile develops a multi-protein classifier in liquid biopsies for early detection of esophageal squamous cell carcinoma from a population-based case-control study. biomark Res. (2021) 9:12. doi: 10.1186/s40364-021-00266-z 33597040 PMC7890600

[14] Yu J Ploner A Kordes M Lohr M Nilsson M de Maturana MEL . Plasma protein biomarkers for early detection of pancreatic ductal adenocarcinoma. Int J Cancer. (2021) 148:2048–58. doi: 10.1002/ijc.33464 33411965

[15] Zhang G Yuan J Pan C Xu Q Cui X Zhang J . Multi-omics analysis uncovers tumor ecosystem dynamics during neoadjuvant toripalimab plus nab-paclitaxel and S-1 for esophageal squamous cell carcinoma: a single-center, open-label, single-arm phase 2 trial. EBioMedicine. (2023) 90:104515. doi: 10.1016/j.ebiom.2023.104515 36921563 PMC10024111

[16] Ferri V Vicente E Quijano Y Duran H Diaz E Fabra I . Predicting treatment response and survival in rectal cancer: insights from 18 FDG-PET/MRI post-neoadjuvant therapy. Int J Colorectal Dis. (2025) 40:6. doi: 10.1007/s00384-024-04803-8 39757340 PMC11700907

[17] Guren AK Baskan Z Balaban Genc ZC Bulun Akyol T Kocaaslan E Agyol Y . The role of baseline PET/CT parameters in predicting treatment response in patients with locally advanced rectal cancer undergoing total neoadjuvant therapy. Medicina. (2025) 61:1449. doi: 10.3390/medicina61081449 40870493 PMC12388065

[18] Nie K Shi L Chen Q Hu X Jabbour SK Yue N . Rectal cancer: assessment of neoadjuvant chemoradiation outcome based on radiomics of multiparametric MRI. Clin Cancer Research: Off J Am Assoc For Cancer Res. (2016) 22:5256–64. doi: 10.1158/1078-0432.ccr-15-2997 27185368 PMC10916000

[19] Zhang L Li L Ren Z Niu Y Lei X Zhang J . Added value of 3D APT histogram analysis to DWI for predicting complete response to neoadjuvant chemoradiotherapy in locally advanced rectal cancer. Eur J Radiol. (2026) 194:112478. doi: 10.1016/j.ejrad.2025.112478 41115362

[20] Lan YQ Hua MW Peng S Wang XL Pan ZC Lin FY . Total neoadjuvant therapy with six versus four cycles of CAPOX in locally advanced rectal cancer: a real-world study. Int J Cancer. (2026) 158:1031–41. doi: 10.1002/ijc.70151 41099620

[21] Guegan JP Legembre P . Nonapoptotic functions of Fas/CD95 in the immune response. FEBS J. (2018) 285:809–27. doi: 10.1111/febs.14292 29032605

[22] Wallach-Dayan SB Petukhov D Ahdut-HaCohen R Richter-Dayan M Breuer R . sFasL-the key to a riddle: immune responses in aging lung and disease. Int J Mol Sci. (2021) 22:2177. doi: 10.3390/ijms22042177 33671651 PMC7926921

[23] Le Bouteiller P Tabiasco J Polgar B Kozma N Giustiniani J Siewiera J . CD160: a unique activating NK cell receptor. Immunol Lett. (2011) 138:93–6. doi: 10.1016/j.imlet.2011.02.003 21324341

[24] Piotrowska M Spodzieja M Kuncewicz K Rodziewicz-Motowidlo S Orlikowska M . CD160 protein as a new therapeutic target in a battle against autoimmune, infectious and lifestyle diseases. Analysis of the structure, interactions and functions. Eur J Med Chem. (2021) 224:113694. doi: 10.1016/j.ejmech.2021.113694 34273660

[25] Hu T Zhang Y Yang T He Q Zhao M . LYPD3, a new biomarker and therapeutic target for acute myelogenous leukemia. Front Genet. (2022) 13:795820. doi: 10.3389/fgene.2022.795820 35360840 PMC8963240

[26] Hu YD Wu K Liu YJ Zhang Q Shen H Ji J . LY6/PLAUR domain containing 3 (LYPD3) maintains melanoma cell stemness and mediates an immunosuppressive microenvironment. Biol Direct. (2023) 18:72. doi: 10.1186/s13062-023-00424-3 37924160 PMC10623712

[27] Huang F Ren Y Hua Y Wang Y Li R Ji N . m6A-dependent mature miR-151-5p accelerates the Malignant process of HNSCC by targeting LYPD3. Mol BioMed. (2024) 5:27. doi: 10.1186/s43556-024-00189-9 39009906 PMC11250566

[28] Xin T Zheng C Li GZ Xu X Zhang J Jia C . Comprehensive analysis of exosome gene LYPD3 and prognosis/immune cell infiltration in lung cancer. Trans Cancer Res. (2024) 13:1394–405. doi: 10.21037/tcr-23-1557 38617517 PMC11009804

[29] Johnsen OF Riis R Meltzer S Augestad KM . Increasing age, neural invasion, extramural vascular invasion, and short-course radiotherapy in locally advanced rectal cancer are associated with decreased tumor regression: a retrospective cohort study. Techniques Coloproctology. (2025) 29:152. doi: 10.1007/s10151-025-03180-w 40715570 PMC12301277

[30] Ng JC Sileo A Sassun R Aboelmaaty S Violante T Gomaa IA . Predictors of pathologic non-response to neoadjuvant approaches in locally advanced rectal cancer. Ann Surg Oncol. (2025) 32:3089–97. doi: 10.1245/s10434-025-16962-1 39920528

[31] Wang Y Pan Z Li S Cai H Huang Y Zhuang J . Prediction and validation of pathologic complete response for locally advanced rectal cancer under neoadjuvant chemoradiotherapy based on a novel predictor using interpretable machine learning. Eur J Surg Oncol J Eur Soc Surg Oncol Br Assoc Surg Oncol. (2024) 50:108738. doi: 10.1016/j.ejso.2024.108738 39395242

[32] Akiyoshi T Shinozaki E Maeda Y Taguchi S Chino A Hanaoka Y . ctDNA longitudinal analysis during total neoadjuvant therapy and nonoperative management for locally advanced rectal cancer: a biomarker study from the NOMINATE trial. Clin Cancer Research: Off J Am Assoc For Cancer Res. (2025) 31:5188–97. doi: 10.1158/1078-0432.ccr-25-1242 40772855

[33] Chang L Zhang X He L Ma Q Fang T Jiang C . Prognostic value of ctDNA detection in patients with locally advanced rectal cancer undergoing neoadjuvant chemoradiotherapy: a systematic review and meta-analysis. Oncologist. (2023) 28:e1198–e208. doi: 10.1093/oncolo/oyad151 37294663 PMC10712909

[34] Wada Y Shimada M Morine Y Ikemoto T Saito Y Zhu Z . Circulating miRNA signature predicts response to preoperative chemoradiotherapy in locally advanced rectal cancer. JCO Precis Oncol. (2021) 5:PO.21.00015. doi: 10.1200/po.21.00015 34913022 PMC8668014

[35] Wang Y Yang L Bao H Fan X Xia F Wan J . Utility of ctDNA in predicting response to neoadjuvant chemoradiotherapy and prognosis assessment in locally advanced rectal cancer: a prospective cohort study. PloS Med. (2021) 18:e1003741. doi: 10.1371/journal.pmed.1003741 34464382 PMC8407540

[36] Liang XJ Guo X Zhu PH Zhao ZH You Z Yang K . An enhanced cancer immunogenic cell death strategy: fibrate-oxaliplatin(IV) conjugates with modulating tumor immune microenvironment. J Med Chem. (2025) 68:26328–47. doi: 10.1021/acs.jmedchem.5c02436 41361896

[37] Png S Tadepalli S Graves EE . Radiation-induced immune responses from the tumor microenvironment to systemic immunity. Cancers. (2025) 17:3849. doi: 10.3390/cancers17233849 41375050 PMC12691506

[38] Tan Y Yang T Jiang S Li S Cai L Wang Y . Oxaliplatin-artesunate conjugate intensifies suppression on colorectal cancer by boosting antitumor immunity. J Inorg Biochem. (2026) 277:113212. doi: 10.1016/j.jinorgbio.2026.113212 41520444

[39] Zhang L Liu Y Yang H Liu L Wang L Chai J . Effect of OASL on oxaliplatin-induced immunogenic cell death in gastric cancer via the cGAS-STING signaling pathway. Cell Death Discov. (2025) 12:20. doi: 10.1101/2024.12.09.627497 41271618 PMC12804755

[40] Zhao S Sun D Yu H Wang M Xu B Wang Y . Oxaliplatin accelerates immunogenic cell death by activating the cGAS/STING/TBK1/IRF5 pathway in gastric cancer. FEBS J. (2025) 292:3814–28. doi: 10.1111/febs.70102 40260556

